# Signal transducer and activator of transcription 5B deficiency due to a novel missense mutation in the coiled-coil domain

**DOI:** 10.1016/j.jaci.2018.08.032

**Published:** 2019-01

**Authors:** Meghan J. Acres, Florian Gothe, Angela Grainger, Andrew J. Skelton, David J. Swan, Joseph D.P. Willet, Suzy Leech, Sonya Galcheva, Violeta Iotova, Sophie Hambleton, Karin R. Engelhardt

**Affiliations:** a Primary Immunodeficiency Group, Institute of Cellular Medicine, Newcastle University, Newcastle upon Tyne, United Kingdom; b Department of Pediatrics, Dr von Hauner Children's Hospital, Ludwig-Maximilians-Universität München, Munich, Germany; c Bioinformatics Support Unit, Newcastle University, Newcastle upon Tyne, United Kingdom; d Great North Children's Hospital, Newcastle upon Tyne Hospitals NHS Foundation Trust, Newcastle upon Tyne, United Kingdom; e Department of Pediatrics, Medical University – Varna, Pediatric Endocrinology, University Hospital “St Marina”, Varna, Bulgaria

To the Editor:

We report a 17-year-old boy who presented with growth hormone (GH)-refractory growth failure (Fig 1, *A*), severe eczema (Fig 1, *B*), and autoimmunity. He was born to consanguineous parents at term with normal gestational weight (3.4 kg) and length (51 cm). He initially presented with autoimmune thyroiditis at age 4 years, with symptoms of lethargy, poor growth, delayed bone age, and constipation (Fig 1, *C*), confirmed by high titers of antithyroid peroxidase antibodies (>1000 IU/L; normal range, <34 IU/L) and antithyroglobulin antibodies (680 IU/L; normal range, <100 IU/L). He was treated with L-thyroxine (levothyroxine), which led to some improvement in his weight and growth. He also suffered from iron-deficiency anemia, which was treated with iron (III) hydroxide polymaltose (Maltofer), and ichthyosis from infancy, and developed atopic dermatitis at the age of 7 years. At age 9 years, he lost his head-hair, eyebrows, and eyelashes, and alopecia persisted despite treatment with steroids (prednisolone) on several occasions; this also failed to resolve his ongoing dermatitis. Three years later he was started on a gluten-free diet after antigliadin antibodies were detected in his blood; however, he had no anti–tissue transglutaminase antibodies and did not meet the clinical criteria of celiac disease. Furthermore, because there was no symptomatic improvement, the gluten-free diet was stopped after 2 years. Meanwhile, he maintained poor linear growth and delayed puberty. He had normal concentrations of basal GH, but low levels of insulin-like growth factor-1 (IGF-1) (Fig 1, *D*), IgA (see Table E1 in this article's Online Repository at www.jacionline.org), and vitamin D. In 3 GH provocation tests he had peak GH levels of less than 10 ng/mL (insulin, 7.35 ng/mL; glucagon, 3.17 ng/mL; arginine, 4.88 ng/mL), which is a response characteristic rather of GH deficiency than of GH insensitivity. However, therapeutic trial with GH for 2 years did not improve growth velocity, and IGF-1 remained below the normal limits throughout the follow-up. Treatment with cyclosporine was started when he was 14 years old and improved the eczema (Fig 1, *B*) and total alopecia, with some relapse of skin rash/thickened dry skin and alopecia after 1 year. Now he is in a stable condition on cyclosporine therapy, with greatly improved skin status and progress into puberty, although he is still small for his age. Hypothyroidism is well compensated by L-thyroxine therapy, but he has chronic recurring alopecia.

**Fig 1:**
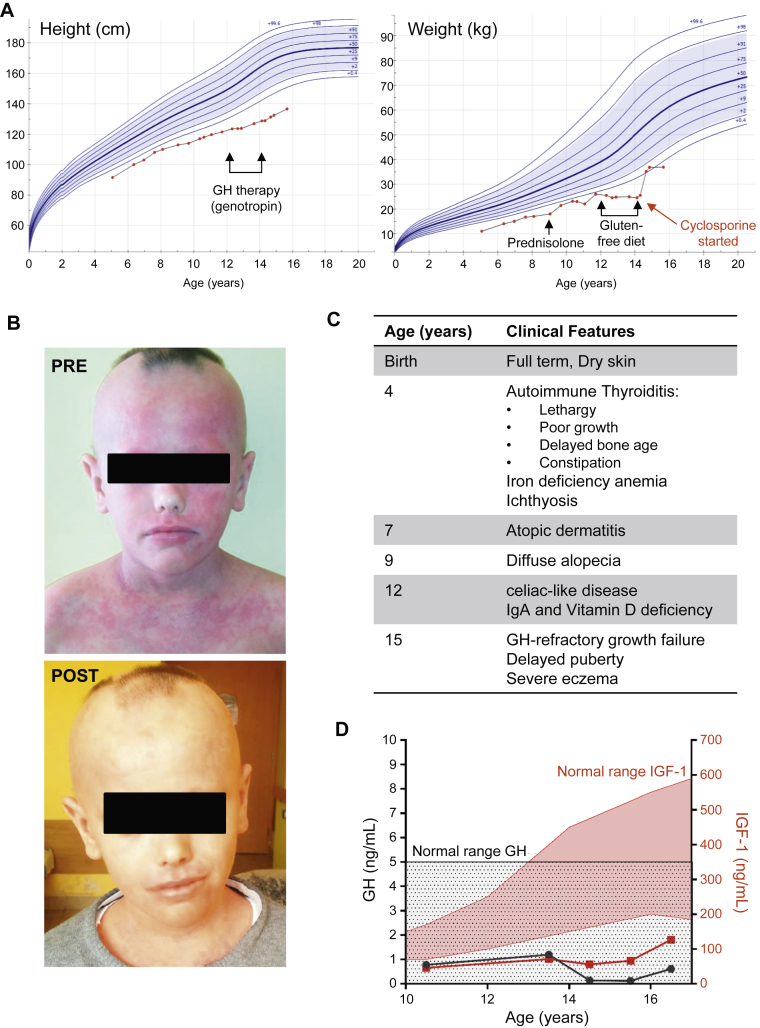
Clinical characteristics. **A,** Height and weight of the patient from age 5 to 15 years. Periods of GH therapy, gluten-free diet, and steroid treatment are indicated by *arrows*. **B,** Eczema and alopecia before (pre) and after (post) cyclosporine treatment. **C,** Patient clinical features. **D,** GH *(black line)* and IGF-1 *(red line)* levels in the patient over time. *Shaded areas*: normal ranges (black, GH; red, IGF-1).

Assessment of his immunological status revealed a slight reduction in naive CD4^+^ T and B cells, a striking increase in class-switched memory B cells, abundant activated HLA-DR^+^ T cells, low levels of IgA and IgM, and increased levels of IgE (Table E1).

In an attempt to identify a presumed autosomal-recessive cause of this striking phenotype, we undertook whole-exome sequencing and found a novel homozygous missense mutation in the coiled-coil domain (CCD) of the gene *STAT5B* (signal transducer and activator of transcription [STAT] 5B; Fig 2, *A*). The nucleotide change c.452T>C, translating to p.L151P, was confirmed by Sanger sequencing and was found to be heterozygous in both parents and wild-type in the healthy sister (Fig 2, *B*). The mutation is predicted to be deleterious by several prediction programs (Fig 2, *C*). Indeed, our patient's phenotype was very compatible with that previously described for STAT5B deficiency. STAT5 functions downstream of cytokines such as IL-2, IL-4, IL-7, IL-9, IL-13, IL-15, IL-21, erythropoietin, thrombopoietin, and GH by translocating to the nucleus upon receptor engagement and activating transcription of growth factors, transcription factors, and other effector molecules. Even though STAT5A and STAT5B share greater than 90% homology, they have nonredundant as well as redundant functions.^1^ The GH receptor preferentially uses STAT5B to induce expression of IGF-I, which promotes skeletal development and adipogenesis.^2^ STAT5B also regulates expression of the transcription factor FOXP3 and the high-affinity IL-2 receptor subunit CD25, both key molecules in the development and function of regulatory T (Treg) cells.^1^ Thus, human STAT5B deficiency is characterized by short stature due to GH insensitivity, and immune dysregulation typically manifest as eczema, chronic diarrhea, recurrent infections, and autoimmune diathesis.^2^ STAT5B-deficient patients have normal levels of GH, but severely reduced serum levels of IGF-I, and are refractory to GH treatment. Other characteristic features are chronic lung diseases such as recurrent pneumonia, lymphocytic interstitial pneumonitis, and/or lung fibrosis, as well as moderate T-cell lymphopenia.^2^, ^3^

**Fig 2:**
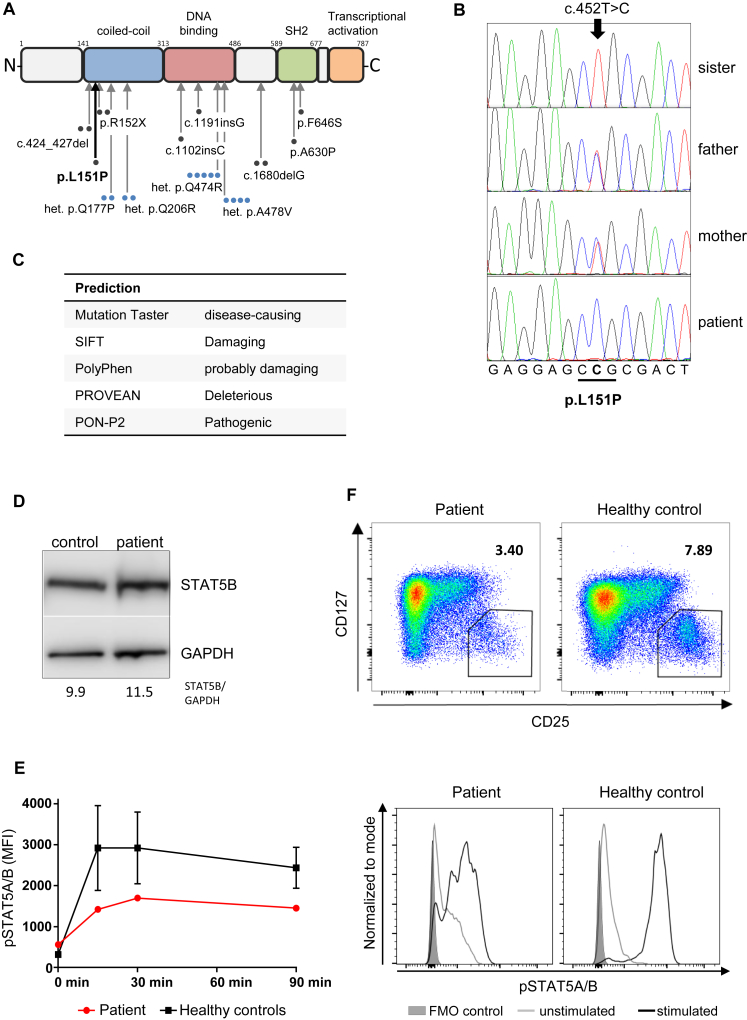
Characterization of L151P. **A,** STAT5B schematic showing L151P in the CCD. *Gray arrows*: previously reported pathogenic mutations. Each *dot* represents 1 individual with either autosomal-recessive (black) or autosomal-dominant (blue) mutations. **B,** Sanger sequencing. **C,** L151P impact predictions. **D,** STAT5B immunoblot, EBV-B cells (loading control: GAPDH). **E,** Phosflow analysis of pSTAT5. PBMCs stimulated with 100 ng/mL IL-2 for 0 to 90 minutes. *Red line*: patient; *black line*: average of 3 healthy controls. Histogram plot: PBMCs gated on live CD3^+^ lymphocytes. *Gray area*: fluorescence-minus-one (FMO) control; *gray line*: unstimulated; *black line*: 15-minute stimulation. **F,** CD4^+^CD25^hi^CD127^−^ Treg cells in patient and control. Numbers indicate percentages of cells within the gate. *GAPDH*, Glyceraldehyde 3-phosphate dehydrogenase.

Since the first mutation in STAT5B was reported in 2003, 9 more patients with autosomal-recessive^2^ and 13 individuals with autosomal-dominant^4^, ^5^ STAT5B deficiency were identified (Fig2, *A*). Homozygous mutations were loss-of-function, with 5 frameshift or nonsense mutations and 2 missense mutations in the SH2 domain. Both of these missense mutations cause aberrant folding of STAT5B, leading to protein aggregation, reduced expression levels, and impaired transcriptional activity.^6^, ^7^ AD STAT5B deficiency with variable penetrance was caused by heterozygous missense mutations in the CCD or the DNA-binding domain, with intact expression of a mutant protein with dominant-negative activity (Fig 2, *A*; see Table E2 in this article's Online Repository at www.jacionline.org).^4^, ^5^ The CCD is important for protein-protein interactions and nuclear import.^8^

To explore the pathogenicity of our patient's p.L151P mutation, we first checked for STAT5B protein expression by immunoblotting, showing normal levels in patient EBV-B cells compared with control (Fig 2, *D*). To test whether the mutant STAT5B is functionally active, we stimulated PBMCs from the patient and healthy controls with recombinant human IL-2 for 0 to 90 minutes and measured STAT5 phosphorylation by flow cytometry. Compared with 3 controls, the patient showed reduced STAT5 phosphorylation (Fig 2, *E*). Because the antibody used recognizes phosphorylated STAT5A and STAT5B, it is possible that the remaining signal entirely reflects phosphorylated STAT5A. A similar result was obtained using patient and control EBV-B cells (data not shown). We next investigated Treg-cell numbers and function, which have been shown to be reduced in patients with STAT5B deficiency.^2^ Compared with the travel control, the CD4^+^CD25^hi^CD127^−^ Treg-cell population was reduced by about half in the patient's peripheral blood (Fig 2, *F*). Treg-cell suppressive capacity was assessed with Treg cells from the patient and a travel control (blood shipped for 36 hours) and an in-house control (fresh blood) (see Fig E1, *A*, in this article's Online Repository at www.jacionline.org). Patient's Treg cells showed significantly reduced suppressive capacity compared with those of the in-house control (*P* = .0021), but not the travel control (Fig E1, *B*). Thus, unfortunately, no conclusion about Treg-cell function can be drawn. T-cell proliferation to all mitogens measured was in the normal range (Fig E1, *C*), albeit perhaps a lower response in the patient was more pronounced for IL-2 (21,894 counts per minute patient vs 47,107 counts per minute control) than for other mitogens. The significance of this finding is not clear, but could relate to a defect in STAT5B signaling downstream of the IL-2 receptor.

How a missense mutation in the CCD affects phosphorylation of Y694 in the transcriptional activation domain is interesting to consider. It has been shown for Stat3 that the α1 helix of the CCD, through intramolecular interactions, is crucial for IL-6–induced recruitment of Stat3 to the IL-6 receptor and subsequent Stat3 phosphorylation, nuclear translocation, and DNA binding.^9^ Because of high structural conservation between STAT proteins, a similar mechanism might apply to the role of STAT5B's CCD in the recruitment of STAT5B to the IL-2 receptor and subsequent phosphorylation. Interestingly, another STAT5B CCD mutation, Q206R, also inhibits IL-2–induced STAT5B-Y694 phosphorylation.^4^ This heterozygous mutant has a dominant-interfering effect on STAT5B transcriptional function and leads to a phenotype of autoimmunity, lymphoproliferation, granulocytosis, and hypogammaglobulinemia (patient 1), or multiple sclerosis, arthritis, and recurrent infections (patient 2), but no GH insensitivity, delayed puberty, eczema, or pulmonary disease (see Table E2 in this article's Online Repository at www.jacionline.org).^4^ In contrast, a further heterozygous STAT5B CCD mutant, Q177P, is robustly phosphorylated, but exerts a dominant-negative effect by abrogating STAT5B nuclear import.^5^ This mutation results in GH-insensitive growth failure, delayed puberty, reduced IGF-1 levels, and severe eczema, as seen in our L151P patient and the 4 patients with nonsense and frameshift mutations in the CCD.^2^, ^5^ Both our patient and the Q177P index patient had normal instead of elevated levels of prolactin, and raised IgE levels were found in both Q177P and 1 R152X patient (Table E2).

In summary, this is the first report of a homozygous hypomorphic missense mutation in the STAT5B CCD leading to functional defects and a phenotype of STAT5B deficiency. This included GH-refractory growth failure, severe eczema, and autoimmune disease but to date without pulmonary problems, severe infections, or T-cell lymphopenia. It would have been ideal to treat the GH insensitivity with IGF-1, but unfortunately, that was not available for the patient. We observed a gratifying clinical response of the atopic dermatitis to cyclosporine therapy, coinciding with improved nutritional status, growth, and general well-being. However, the long-term outlook remains guarded. Alternative treatment strategies for immune dysregulation one might consider if symptoms deteriorated include sirolimus therapy or even stem cell transplantation.

For detailed methods, please see the Methods section in this article's Online Repository at www.jacionline.org.

## Figures and Tables

**Fig E1:**
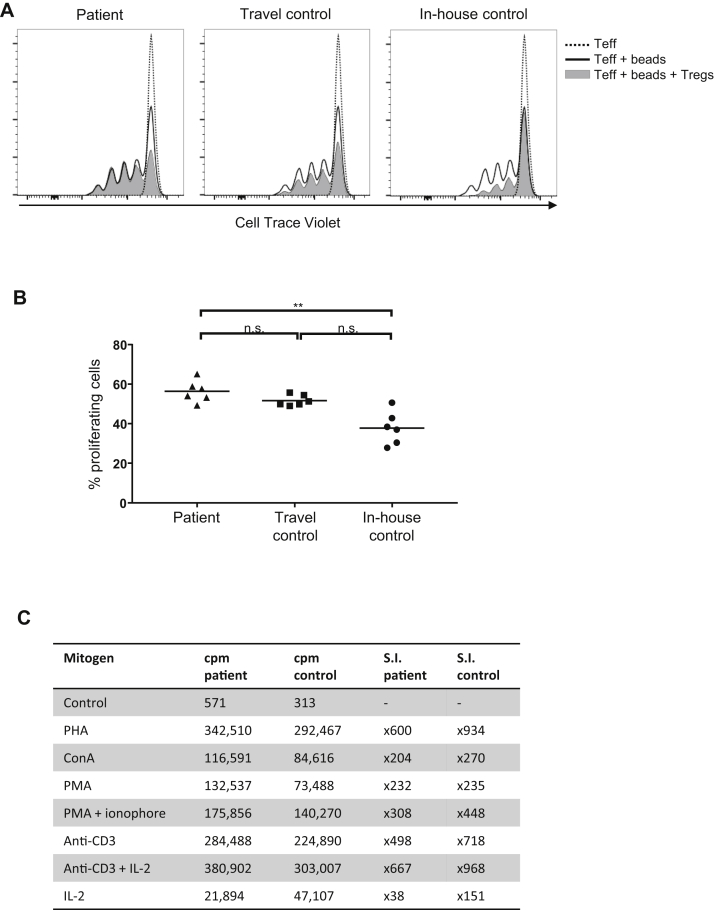
**A,** Treg-cell suppression assay. *Dotted line*: Teff alone. *Solid line*: Teff stimulated with Treg-cell inspector beads. *Shaded area*: Teff stimulated with Treg-cell inspector beads in the presence of Treg cells. Proliferation of Cell trace violet-labeled Teffs was measured as dilution of the dye with every cell division. Inhibition of Teff proliferation by Treg cells is shown by reduced dye dilution. Results are shown for the patient, a travel control (both performed with 36-hour-old blood), and an in-house control (fresh blood). **B,** Percentage of proliferating cells with means of 6 replicates given. Patient's Treg cells showed significantly reduced suppressive capacity compared with those of the in-house (**Kruskal-Wallis-Test: *P* = .0021) but not those of the travel control. **C,** Lymphocyte proliferation, measured as counts per minute (cpm) of incorporated tritiated thymidine, was normal to all mitogens tested. *ConA*, Concanavalin A; *n.s.*, nonsignificant; *PMA*, phorbol myristate acetate; *S.I.*, stimulation index.

**Table E1:** Laboratory evaluation of STAT5B^L151P^ patient

Parameter	Patient	Reference range∗ (11-14 y)
Neutrophils	2810 cells/μL	1500-8000 cells/μL
Lymphocytes	3170 cells/μL	1700-6900 cells/μL
CD3^+^	2995 cells/μL	900-4500 cells/μL
CD4^+^	2315 cells/μL	500-2400 cells/μL
CD8^+^	633 cells/μL	300-1600 cells/μL
CD19^+^	270 cells/μL	200-2100 cells/μL
CD27^−^IgM^+^IgD^+^ (naive)	51%	75%-87%
CD27^+^IgM^+^IgD^+^ (memory)	9%	5%-10%
CD27^+^IgM^−^IgD^−^ (class switch memory)	30%	3%-10%
CD4^+^/CD45RA^+^/CD27^+^ (naive)	240 cells/μL (8%)	300-800 cells/μL
CD4^−^/CD45RA^+^/CD27^+^ (naive)	180 cells/μL (6%)	150-400 cells/μL
CD4^−^/CD45RA^+^/CD27^−^ (effector)	60 cells/μL (2%)	<60 cells/μL
Activated T cells (HLA-DR^+^)	56%	<30%
NK cells (CD16^+^/CD56^+^)	205 cells/μL (6%)	70-1200 cells/μL
IgA	0.1 g/L	0.14-1.23 g/L
IgM	0.43 g/L	0.48-1.68 g/L
IgG	10.1 g/L	4.24-10.51 g/L
IgE	491 IU/mL	0-200 IU/mL
Tetanus antibody	0.31 IU/mL	0.1-10 IU/mL
Hemophilus B antibody	0.2 mg/L	1-20 mg/L
Pneumococcal serotypes: protective responses	8/12 serotypes	
Measles IgG	Detected	
Varicella zoster IgG	Detected	
Toxoplasma IgG	Detected	
HIV screen	Negative	
Hepatitis screen	Negative	
IGF-1	62.5 ng/mL	143-693 ng/mL
TSH	4.72 μIU/mL	0.4-4 μIU/mL
Prolactin	95.4 μIU/ml	53-360 μIU/mL

*NK*, Natural killer.

∗ In-house reference range.

**Table E2:** Comparative table of CCD mutations in STAT5B

Characteristic	L151P (n = 1)	Q177P (n = 2)	Q206R (n = 2)	R152X (n = 2)	c.424_427del (n = 2)
Mutation	Homozygous missense	Heterozygous missense	Heterozygous missense	Homozygous nonsense	Homozygous indel, frameshift (p.L142fsX161)
STAT5B protein expression	Normal	Normal	Normal	Absent	Unknown (expected: absent)
STAT5 phosphorylation	Reduced upon IL-2 stimulation of PBMCs	Increased upon GH and IFN-γ stimulation of fibroblasts; defective nuclear import of STAT5B	Reduced upon IL-2, IL-7, or IL-15 stimulation of T-cell blasts	Reduced upon IL-2 stimulation of PBMCs	Unknown
Treg cells	Reduced numbers of CD4^+^CD25^hi^CD127^−^ Treg cells	Unknown	Normal frequency of CD4^+^FoxP3^+^ Treg cells, diminished CD25 expression (1/1)	Reduced numbers of CD4^+^CD25^hi^ Treg cells (1/1)	Unknown
Postnatal growth failure	Yes	Yes (2/2)	No	Yes (2/2)	Yes (2/2)
Delayed puberty	Yes	Yes (2/2)	No	Yes (2/2)	Yes (1/1)
Basal GH (ng/mL) [<10 ng/mL]	Normal (0.6-1.2)	Normal (1/1) (0.4)	Unknown	Normal (2/2) (6.6; P1) (1.8; P2)	Normal (2/2) (1.7; P1) (1; P2)
Basal IGF-1 (ng/mL) [95-400]	Reduced (46-126)	Reduced (2/2) (56; P1) (58; P2)	Unknown	Not detectable (2/2)	Reduced (2/2) (34; P1) (<25; P2)
IgE	Raised	Raised (2/2)	Unknown	Raised (1/1)	Normal (2/2)
Prolactin	Normal	Normal (1/1)	Unknown	Raised (1/1)	Raised (2/2)
Eczema	Severe	Severe (1/1)	No	Severe (2/2)	Present (2/2)
Autoimmunity	Autoimmune thyroiditis, alopecia	No	Immune thrombocytopenic purpura (P1)	No (P1) Autoimmune thyroiditis (P2)	No (P1) Thrombocytopenic purpura (P2)
Chronic pulmonary disease	No	Yes (1/1)	No	Yes (2/2)	Yes (2/2)
Viral infections	No	Unknown	EBV	VZV (2/2)	Unknown
Others	Celiac-like disease Iron-deficiency anemia Vitamin D deficiency Reduced IgA	Asthma	Splenomegaly, lymphadenopathy, necrotizing granulomas/granulocytosis Hypogammaglobulinemia (P1) Multiple sclerosis, arthritis, recurrent infections (P2)	Chronic diarrhea	
Reference		^E5^	^E6^	E7, E8	^E9^

*P1*, Patient 1; *P2*, patient 2; *(1/1)*, 1 of 1 patient analyzed; *(2/2)*, 2 of 2 patients analyzed.

## Methods

### Study subjects

Patients and their relatives provided written informed consent to participate in research protocols approved by the Newcastle and North Tyneside 1 Research Ethics Committee. Whole blood samples were obtained from these individuals, and genomic DNA was isolated using the DNeasy or QIAamp DNA mini kit (Qiagen, Manchester, UK).

### PCR and sequencing analysis

Specific primers were designed in Primer3web version 4.0.0 (http://bioinfo.ut.ee/primer3/). Primer sequences are available on request. Capillary sequencing was performed according to standard methods. Sequences were aligned with the consensus coding sequence (human genome assembly 38) in nucleotide BLAST (http://blast.ncbi.nlm.nih.gov/blast/). ChromasLite Version 2.1.1 was used for visualization of the sequences.

### Whole-exome sequencing

Whole exome of the patient was captured using the Agilent SureSelect V5 kit and a library subsequently prepared for paired-end sequencing. This was carried out on an Illumina NextSeq instrument, on a high-output flowcell by OGT (Oxford Gene Technology, Begbroke, UK). Sequencing reads were assessed for quality using the FastQC and MultiQC tools.E1, E2 Reads were aligned to the hg19 assembly of the human reference genome, and prepared for variant calling. Exomes were analyzed according to GATK best practices,^E3^ as follows: the raw alignment was realigned around indels, quality scores recalibrated (Base Quality Score Recalibration [BQSR]), and duplicates marked using the Picard suite. Genotyping was performed using a combination of the HaplotypeCaller from GATK in the gVCF mode, and JointCalling program leveraging a catalogue of exome samples from other patients with primary immunodeficiency. To avoid false-positive calls, Variant Quality Score Recalibration (VQSR) was performed on the raw variant callsets for indels and single nucleotide polymorphisms. Downstream analysis was carried out using the Web-based analytics application Ingenuity Variant Analysis (Qiagen).

### Cell lines

EBV-immortalized B-cell lines were generated from PBMCs after Ficoll-purification from whole blood (EDTA). Cells were incubated with EBV-containing medium for 1 hour initially, and then in the presence of 1 μg/mL cyclosporine A for a further 24 hours at 37°C, 5% CO_2_. Cells were then transferred to fresh 10% FCS/cyclosporine A–containing RPMI and incubated until foci of immortalized cells appeared.

### Immunoblotting

Cell lysates were prepared from patient and control EBV-B cells. Equal amounts of lysate were separated by electrophoresis on a 4% to 12% polyacrylamide gradient gel in 3-(N-morpholino)propanesulfonic acid buffer (NuPAGE; Novex, Fisher Scientific, Loughborough, UK) and transferred to a polyvinylidene fluoride or polyvinylidene difluoride membrane (Immobilon-P; Millipore, Watford, UK). STAT5B was probed using rabbit-anti-human STAT5B mAb (EPR16671, Abcam, Cambridge, UK) and anti-rabbit IgG, horseradish peroxidase–linked secondary antibody (Cell Signalling Technology [London, UK], 7074P2). Anti–glyceraldehyde 3-phosphate dehydrogenase antibody (Cell Signalling Technology, 5174S) was used as a loading control.

### Flow cytometry

Whole blood (EDTA) was rested for 2 hours at room temperature and then stimulated for 0, 15, 30, and 90 minutes at 37°C with 0.1 μg/mL recombinant human IL-2 (Peprotech [London, UK], 200-02). Alternatively, EBV-B cells were serum-starved for 1 hour and then stimulated with 0.5 μg/mL recombinant human IL-2. Whole blood cells were stained for the surface marker CD3 and intracellularly for phosphorylated STAT5 (pSTAT5), EBV-B cells for pSTAT5 only. Samples were acquired using a FACSCanto II cytometer (BD Biosciences [BD], Wokingham, UK) and analyzed with FlowJo (Treestar, Ashland, Ore). The following antibodies were used: Pacific Blue mouse anti-human CD3 (clone UCHT1, BD Biosciences) and Alexa Fluor 647 mouse anti-human STAT5 (pY694) (clone 47, BD Biosciences).

### Lymphocyte proliferation

PBMCs were cultured for 3 days with the respective mitogens, and proliferative responses were measured as counts per minute of tritiated thymidine incorporated during cell division. Cultures were performed in triplicate, with results expressed as the means of the triplicates. The stimulatory index is calculated as stimulated over unstimulated (control) counts.

### Treg-cell suppression assay

CD4^+^ T cells were enriched using RosetteSep (Stemcell Technologies, Cambridge, UK). To isolate CD4^+^CD25^−^ effector T cells (Teff), CD25 microbeads and an LD column were used (both Miltenyi Biotec) on a fresh healthy control sample. The Teff cells were labeled with Cell trace violet (Invitrogen, Thermo Fisher Scientific, Loughborough, UK) and stimulated with anti-CD2/CD3/CD28-human Treg-cell inspector beads (Miltenyi Biotec, Woking, UK) for 4 days. Treg cells from the patient, a travel control (36-hour old blood samples), and an in-house control (fresh blood sample) were purified via flow sorting (FACS Fusion, BD) gated on CD4^+^CD25^hi^CD127^−^ cells. These cells were added at a ratio of 1:1 to the Teff population during the stimulation period. Proliferating Teff cells were evaluated using a FACS Canto flow cytometer (BD). Cultures were performed in sextuplicates.
